# Development of a Pattern Recognition Methodology with Thermography and Implementation in an Experimental Study of a Boiler for a WHRS-ORC

**DOI:** 10.3390/s19071680

**Published:** 2019-04-09

**Authors:** Concepción Paz, Eduardo Suárez, Miguel Concheiro, Antonio Diaz

**Affiliations:** School of Industrial Engineering, University of Vigo, Campus Universitario Lagoas-Marcosende, 36310 Vigo, Spain; suarez@uvigo.es (E.S.); mconcheiro@uvigo.es (M.C.); adiaz@uvigo.es (A.D.)

**Keywords:** WHRS, ORC, boiler, infrared thermography

## Abstract

Waste heat dissipated in the exhaust system in a combustion engine represents a major source of energy to be recovered and converted into useful work. A waste heat recovery system (WHRS) based on an Organic Rankine Cycle (ORC) is a promising approach, and it gained interest in the last few years in an automotive industry interested in reducing fuel consumption and exhaust emissions. Understanding the thermodynamic response of the boiler employed in an ORC plays an important role in steam cycle performance prediction and control system design. The aim of this study is, therefore, to present a methodology to study these devices by means of pattern recognition with infrared thermography. In addition, the experimental test bench and its operating conditions are described. The methodology proposed identifies the wall coordinates, traces the paths, and tracks the wall temperature along them in a way that can be exported for subsequent post-processing and analysis. As for the results, through the wall temperature paths on both sides (exhaust gas and working fluid), it was possible to quantitatively estimate the temperature evolution along the boiler and, in particular, the beginning and end of evaporation.

## 1. Introduction

Increasingly restrictive international regulations concerning greenhouse gas emissions demand the development of cleaner and more efficient internal combustion engines (ICEs). Most modern diesel engines are achieving a maximum of about 35% in thermal efficiency—the remaining heat is simply wasted. Waste heat is dissipated through three main channels: the coolant system, convection and radiation from the engine block, and—most importantly—the exhaust system, which alone accounts for up to 40% of total fuel energy [1,2,3,4]. 

In the last few years, the automotive industry showed great interest in waste heat recovery systems (WHRS) based on an Organic Rankine Cycle (ORC) because of the potential for recovering some of that energy. This would achieve significant reductions in fuel consumption and, as a result, exhaust emissions [4,5,6]. This technology was already widely implemented over the past decade in the marine sector [7,8]; however, reducing the components of the cycle to the scale of a passenger vehicle is still a challenge. Nevertheless, there is room to work with in heavy duty vehicles and, thus, work is continuing [9].

Predicting the behavior of a boiler is fundamental in the development and design process of these systems. Most boiler load losses are due to the superheated steam zone; hence, it is very important to estimate its length adequately. In principle, there are correlations that can be used to predict a boiler’s thermal efficiency and head loss. These correlations are different for each section; therefore, determining the location of phase changes in a boiler is essential.

This study sets out to do just that using a test bench with an ORC-WHRS boiler. We study it from two different approaches: on one hand, the monitored sensors retrieve overall parameters of the boiler, such as outlet temperature, pressure drop, and performance; on the other hand, an infrared (IR) thermographic camera captures thermal images of the external walls of the boiler.

IR thermography is a non-contact and non-destructive technique well consolidated and applied in a wide range of fields, such as medicine and the military [10]. There is even some existing research in the literature that studies multiphase heat transfer processes with thermographic analysis. Liu and Pan [11] developed a method to measure the fluid temperature in parallel with the visualization of the two-phase flow pattern in micro channels. Hetsroni et al. [12] used an IR technique to investigate a heat sink for electronics cooling at low heat fluxes and to maintain the temperature of the heated surface at uniform temperatures. Xu et al. [13] carried out boiling heat transfer experiments where an IR high-speed camera was used to measure a chip’s surface temperature and identify transient flow patterns in an array of triangular silicon-based microchannels. Li and Hrnjak [14] presented an IR thermography-based method to quantify the distribution of liquid refrigerant mass flow rate in a parallel flow microchannel heat exchanger. Leblay et al. [15] measured heat transfer coefficients of water flowing in a round tube and in a multiport flat tube. Carlomagno et al. [16] analyzed the ability of IR thermography to perform convective heat transfer measurements and surface visualizations in complex fluid flows.

In the present study, IR thermography was used to experimentally analyze single-phase and multiphase flows involved in the boiler of an ORC. Although the working fluid (WF) is not optically accessible, thermography allows us to quantitatively estimate and understand the fluid dynamic behavior development of both the gas and the working fluid by inferring their properties from the wall temperatures of their adjacent walls. This study is focused on the implementation of a methodology capable of systematically handling a big database of these thermal images, based on a pattern recognition scheme, which automatically detects the boiler and extracts the temperatures to be analyzed.

The rest of this paper is divided in three sections. Section 2 describes the experimental set-up and the boiler of study. Section 3 presents the methodology developed. Section 4 shows and discusses the results obtained, our conclusions, and an overview of future work.

## 2. Experimental Set-Up

The experimental test bench was conceived as a tool to test boilers for implementation in an ORC-based WHRS for the automotive industry. The working fluid must have a high critical temperature, as well as high condensation and evaporation temperatures, suitable for a high-temperature ORC system. Among the fluids that meet these requirements—such as ethanol, R1233zd, siloxane, or *n*-octane—ethanol was chosen because of its low environmental impact and low health risks [17]. Furthermore, when the facilities were designed, ethanol was the working fluid of choice for many automotive manufacturers. 

**Figure 1:**
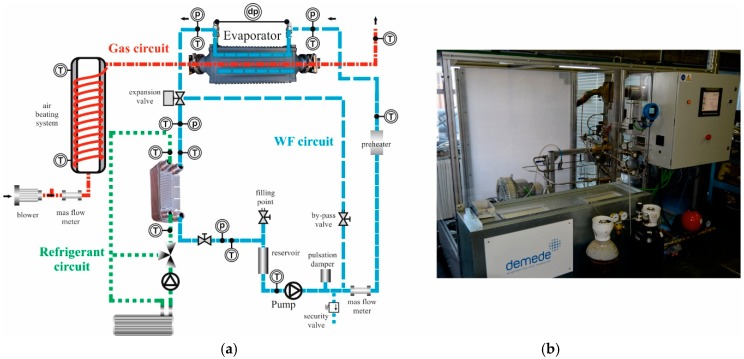
Experimental test bench, schematic diagram (**a**), and photograph (**b**).

The test bench has three circuits: air, ethanol, and water refrigerant. Ethanol is preheated and pumped to the evaporator, where it comes into contact with previously warmed air. After that, the ethanol is cooled in the condenser by transferring its heat to the refrigeration water. Figure 1 shows a scheme of the hydraulic circuit and its characteristics.

The test bench control system is based on a programmable logic controller (PLC). It can be accessed either directly from the test bench or with some complementary software allowing remote access or a Secure Digital (SD) card suite. Ultimately, the software enables the computer and test bench to exchange information by transferring all the data from the bench to the computer in order to analyze it.

The test bench includes a bypass valve system for safe emptying, cleaning, and ethanol dragging, in the event the boiler is replaced to test a different one. The characteristics of the main components of the test bench, with the exception of the boiler itself, which is discussed separately in this section, are listed in Table 1.

Temperature sensors are distributed all along the test bench to provide the air, ethanol, and water temperature at different points of the bench. These points include, among others, the evaporator inlet and outlet and the ethanol preheater inlet and outlet, which allow us to characterize these points’ thermal efficiencies. Pressure drop sensors are mounted on the working fluid side. The main sensors of the experimental set-up are described in Table 2.

The air circuit provides air at a suitable temperature and flow rate to the evaporator after being heated in the heating system. It has three main components: blower, flow rate gauge, and heating system. It basically consists of an open circuit, where air is taken from the environment using an electric turbine as a blower, and a heating system, where the air’s temperature is gradually increased until the required temperature before entering the boiler.

The ethanol circuit is the operation control most prone to instability in the whole system. It is designed to pump the liquid ethanol into the evaporator and then through a condenser, where it is cooled before entering a storage tank being pumped again. The eccentric screw pump pumps the ethanol at the set pressure. Just before entering the boiler, an electric preheater helps the ethanol’s temperature to rise to the set temperature. A plate condenser cools the ethanol after the expansion valve. The storage tank stores all the ethanol after it is cooled in the condenser and keeps it at the proper pressure before entering the pump again.

The experimental bench also has a refrigeration system that cools the hot ethanol leaving the evaporator and further cools the ethanol before entering the storage tank. The components of this circuit are a centrifugal pump that pressurizes the water so that it reaches the condenser, a regulation valve, and a heating unit, which consists of a forced convection air heater. The working fluid for this system is water, which flows through the condenser in counter-current. 

**Figure 2:**
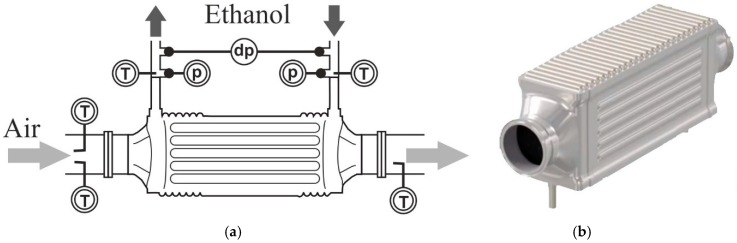
Experimental boiler: scheme (**a**) and photograph (**b**).

The boiler studied was a shell and tube heat exchanger in cross-flow and counter-flow, made entirely of steel AISI 316L. Air flows through a bundle of tubes, while the working fluid flows inside them, in a cross-flow configuration. The working fluid is divided between the first row of tubes, which at the end converge into a mixture chamber to be divided again in the following row. The performance of the boiler was monitored continuously with thermocouples measuring inlet and outlet air and working fluid temperatures. Pressure drop on both sides was recorded by pressure sensors and a differential pressure sensor. The boiler was fully coated, with a double layer of thermal black paint on its external surfaces, in order to make a visual contrast with the background and avoid shiny reflections in the surface of metal, which could introduce errors in the measurements with the thermographic camera. This black coating, with a thickness around 25 μm, increased the emissivity of thermal radiation of the boiler, which according to the specification of the manufacturer was equal to 0.98, an effect that was taken into account later in the discussion. Figure 2 represents a scheme of the boiler studied and a photograph.

Wall temperature was monitored using a thermographic camera Flir E60, by Flir Systems. Some technical characteristics of the camera are shown in Table 3.

## 3. Methodology

The predominant experimental tool used in this study was infrared thermography. The objective of this noninvasive method was to extract a continuous wall temperature profile along the boiler, which provides information about the fluid underneath the walls. This section is divided in two parts: firstly, the experimental procedure is presented (i.e., how the experiments were carried out and what considerations were taken when the thermal images were shot); secondly, the post-processing of the thermal images is presented.

### 3.1. Experimental Procedure

**Figure 3:**
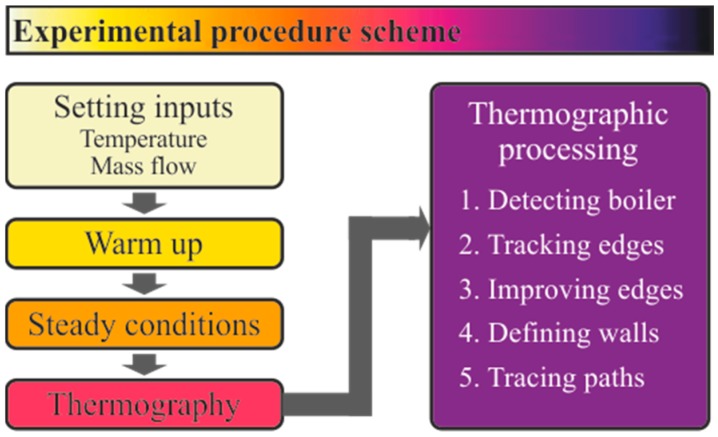
Scheme of the experimental procedure.

The experimental procedure is summarized in Figure 3. To run the experiments at the test bench, the inputs of the circuit were set in the bench software: inlet temperature, pressure, and mass flow rate for both air and ethanol circuits. Firstly, the test bench needed to be warmed up. This was done by gradually introducing the desired values of mass flow and system temperature in both circuits. This was done to ensure a proper functioning and avoid overheating and any damage to the components. The measurements and the thermal images were taken once the monitored outputs confirmed that the test reached a steady state. Subsequent experiments could be performed using the conditions of the previous one, without the need to restart the circuit configuration.

**Figure 4:**
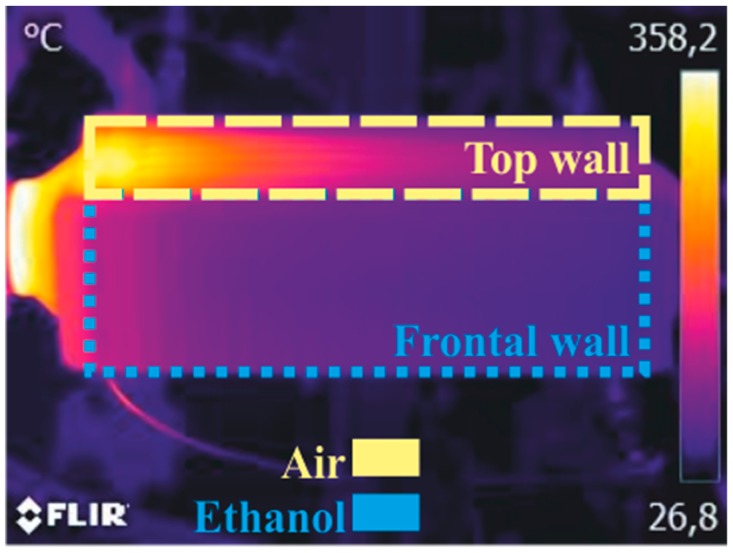
Schematic view of the thermographies.

As shown in Figure 4, from the point of view of the thermal image, the top wall was adjacent to the gas side, whereas the frontal wall was adjacent to the mixing chambers of the working fluid; thus, the thermographic analysis of these walls made it possible to quantitatively learn about thermodynamic behavior of the fluids inside the boiler. Bearing this idea in mind, the relative positioning of the thermographic camera and the boiler made it possible to picture two walls at a time, one adjacent to the fluid and the other adjacent to the gas. Figure 4 shows a schematic view of the perspective of the boiler from the camera.

The images were taken by placing the camera on a tripod at a distance of approximately 50 cm at an angle of about 30° relative to the frontal wall. The thermographic camera used, the model Flir E60, takes pictures with a resolution of 320 × 240 pixels and can measure in a range of temperatures from −20 °C to 650 °C with an accuracy of ±2 °C or ±2% at room temperature and a sensitivity of 0.05 °C.

### 3.2. Thermographic Processing

Raw thermal images were post-processed with the free software Flir Tools. This software can convert the temperature of every pixel into an array of the dimension of the resolution of the camera with the values of temperature in each element. This array was then exported to Matlab in a *.csv file, which performed the calculations needed in this study. The thermal image shows the wall temperature of the boiler, but also the temperatures of the background of the scene. Since these values are not of interest, they were eliminated in the processing.

To do this, a very simple method was tested first. The images were given a temperature limit, and all the pixels (in .csv, a cell, and in Matlab, an element of the array) that did not reach a minimum temperature had their values converted to not a number (NaN) values. Then, pixels were arranged by rows and by columns, with all rows and columns with an average value of NaN lower than a certain value being eliminated. To eliminate the hot tubes that did not completely disappear after the previous filtrations, a filtering process was carried out by columns and rows with a minimum number of non-NaN. This method was quite effective, but in cases where the boiler was inclined, the cutting section was too horizontal, and some data were lost. Another problem was that the perspective was lost and, thus, the construction of the lines in their correct position.

With this in mind, a new methodology was developed. This methodology detects the boiler through a pattern recognition approach. Wall temperature paths were traced and plotted automatically. Wall temperature values in both gas and working fluid regions, both parallel and perpendicular to the flow of the fluids, were tracked. The whole process can be divided in five steps: detecting the boiler, tracking the edges, improving the edges, tracing the walls, and defining the paths. Figure 4 to Figure 8, included in this section, are used to illustrate the methodology, and do not represent any particular experimental point. 

#### 3.2.1. Detecting the Boiler

**Figure 5:**
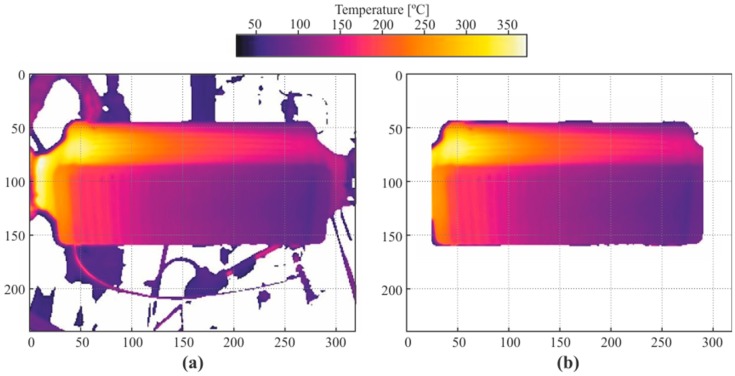
(**a**) Sample of the core of the boiler detected. (**b**) Sample of thermography after filtering cold pixels in the background.

To identify the shape of the boiler, a temperature difference filter was applied, thus taking advantage of the sharp difference between the boiler and room temperature. Some pipes and ducts from the circuit can be seen in the thermal image, but they all belonged to the ethanol circuit, whose temperatures were significantly lower than the temperature of the boiler. To filter these out, the first step was to coarsely discard the pixels belonging to the background. To do so, the pixels of temperature below a threshold of 40 °C were replaced by black pixels, resulting in an image similar to that displayed in Figure 5a. Notice that some components of the circuit with higher temperatures than the threshold temperature are still displayed.

To discard the remaining undesired pixels, the rows and columns of pixels with less than a threshold number of blank pixels were filtered and set as blank pixels too. A sample of a resulting image is displayed in Figure 5b. Note that, to apply the method shown here, the boiler was positioned as horizontally as possible from the perspective of the thermographic camera.

#### 3.2.2. Tracking the Edges

**Figure 6:**
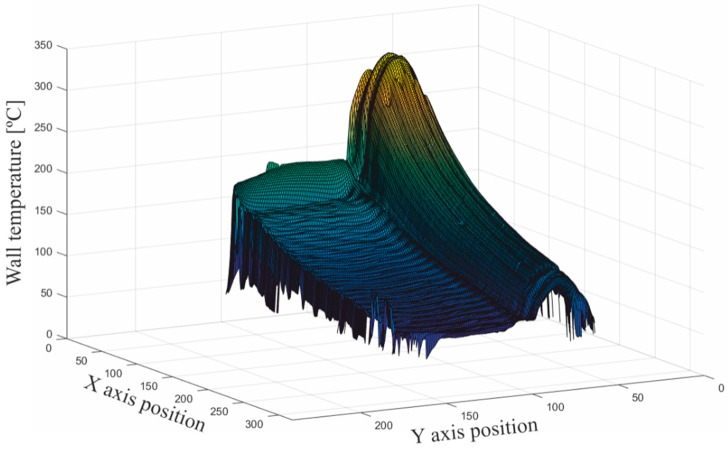
Three-dimensional (3D) graph of the temperatures identified as belonging to the boiler at this step of the procedure.

**Figure 7:**
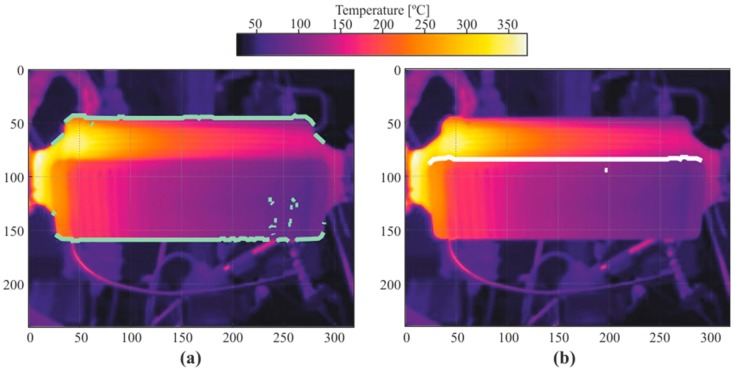
(**a**) The reconstruction of the outermost edges is represented in the color green. (**b**) The reconstruction of the middle edge is represented in the color white.

As shown in Figure 5b, the boundaries of the boiler were not as yet neatly defined. To identify the horizontal edges of the boiler, the highest wall temperature gradient was chosen as the next filter criterion. In Figure 6, a three-dimensional (3D) graph of temperatures of the boiler is plotted. Here, it is clear that the maximum temperature gradient corresponded to the outermost edges of the boiler from the perspective of the camera. The edge between the top wall and the frontal wall became an inflection point of the temperature profiles. Figure 7 shows the reconstruction of the edges of the boiler using this approach. 

#### 3.2.3. Improving the Edges

As shown in Figure 7a, the pixel cloud representing the horizontal edges obtained in the previous step still showed noise and did not seem to be the straight line we know it ought to be. To convert that point cloud into a straight line, two further steps were taken: filtering outliers and a Hough transform [18,19,20].

The outliers were filtered by their deviation from the median of the vertical location of the pixels, when the pixel was an outlier *q* with the following criterion:

(1) $$\begin{array}{ll} & q<Q_{1}-1.5\;IQR,\\ \mathrm{or} & \\ & q>Q_{3}+1.5\;IQR \end{array}$$

where *Q*_1_ and *Q*_3_ are the first and the third quartiles, respectively, and IQR is the interquartile range.

Once the outliers were filtered (Figure 7b), a Hough transform was applied. A Hough transform is a digital image processing technique based on feature recognition. The technique consists of identifying any geometrical instance that can be parametrized, such as straight lines, circumferences, or ellipses. This very effectively converted the remaining point clouds representing the edges into a straight line.

#### 3.2.4. Defining the Walls

Because the output of the Hough transform was a line—and not a geometrical segment—to define the frontal and top walls in the images, the vertices of the now-clear edges had to be found.

There were two issues to solve in this phase. The first was that, although the outlier points should have been filtered by the criterion previously discussed, it was observed that some of these points remained because they belonged to the gas boxes. The second issue was found in the particular boiler used in this study. Due to its manufacturing procedure, the plaque that makes up the superior wall was framed into the gas boxes, deforming it slightly.

Taking these two considerations into account, the vertices of the edges were determined by finding the first point at a distance of more than two pixels from the line previously found with the Hough transform.

**Figure 8:**
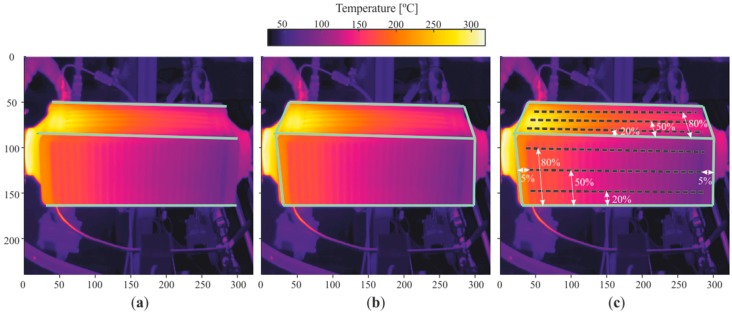
Walls detected by the developed methodology: (**a**) main edges detected, (**b**) walls of interest, (**c**) studied path positions.

Once the horizontal edges on the top and frontal walls were defined (Figure 8a), the remaining vertical edges were defined by tracing a straight line between the vertices of the horizontal edges, thus obtaining the walls of interest, as shown in the graph in Figure 8b.

#### 3.2.5. Tracing Paths

**Figure 9:**
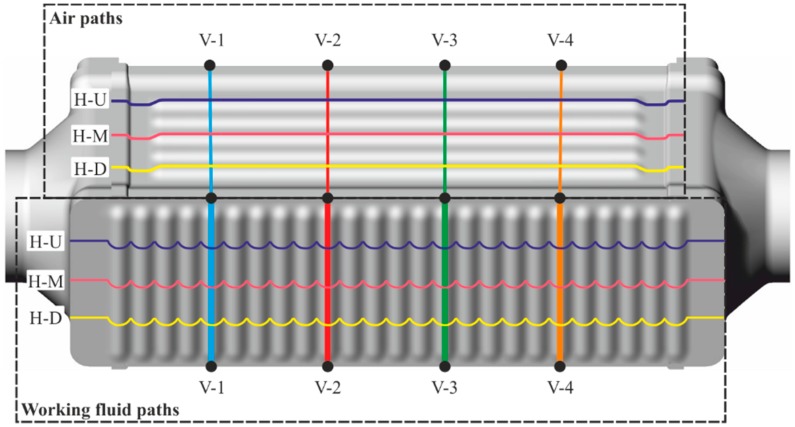
Definition and nomenclature of the paths studied.

Having defined the four edges that define the walls of interest, the path where the properties of wall temperatures were tracked could be defined. Paths of wall temperature were defined on both the frontal and top walls, longitudinal to the flow of both fluids involved. In terms of percentage of the corresponding edge longitude, every path was at 5% away from the vertical edge, and 20%, 50%, and 80% away from the middle edge, as shown in the graph in Figure 8c, and in more detail in Figure 9.

To retrieve the value of the temperature of the paths, the paths were divided into 200 points, and a two-dimensional interpolation was calculated between the two nearest pixels from the image, with the vertical coordinate as a reference. To reduce the oscillations in the values of wall temperature due to the ripples of the external surfaces in the paths of both the gas and working fluid sides, profiles of the horizontal wall temperature paths were smoothed using the robust local regression method with weighted linear least squares and a second-degree polynomial model, which was carried out using MATLAB software.

## 4. Results and Discussion

Throughout the study, we ran the test under different conditions as a control of the methodology. The results of the test carried out under the operating conditions, detailed in Table 4, are discussed in this section.

**Figure 10:**
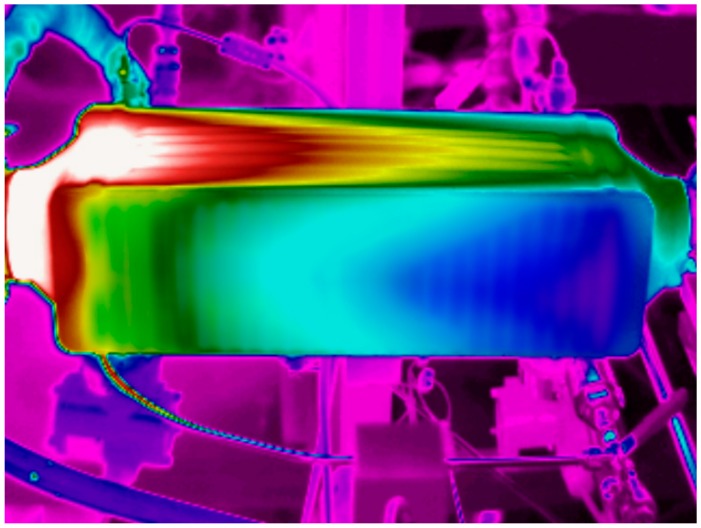
Thermography of the boiler with the operating conditions described in Table 4.

Figure 10 shows the thermal image corresponding to the test point previously described.

**Figure 11:**
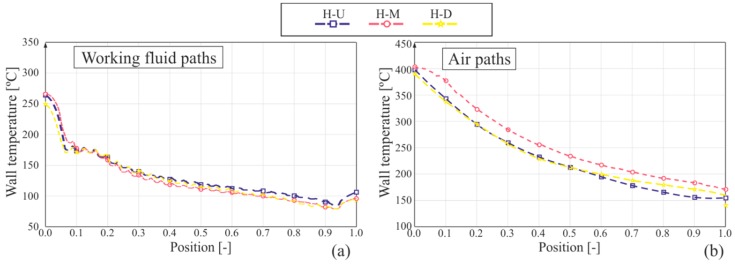
Graphs of horizontal temperature profile: (**a**) working fluid (WF) paths and (**b**) in the air.

The lines in Figure 11a represent the horizontal profiles of wall temperature in the wall adjacent to the mixture chambers on the working fluid side. The slopes of the curves clearly show four stages. From left to right, the first and hottest stage represents the portion of the wall which is adjacent to the gas side. The second stage of the curve shows a portion where there are no great changes in the slope of the curve (in fact, it is almost constant), which indicates a phase change on the working fluid side. In the third stage, the slope remains negative, indicating that the working fluid is being cooled down in a monophasic flow. In the last stage, wall temperature increases again, where the wall is again adjacent to the gas side.

**Figure 12:**
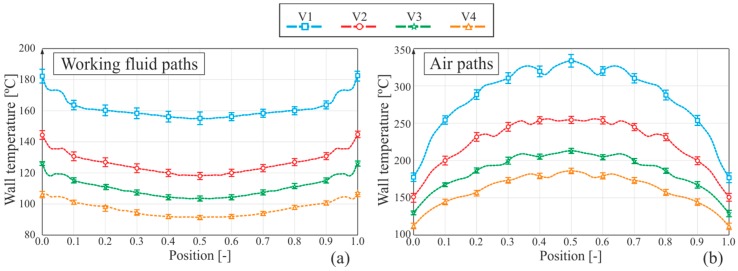
Graphs of vertical temperature profile: (**a**) WF paths and (**b**) in the air.

Figure 11b shows the horizontal paths of wall temperature on the wall adjacent to the gas side, i.e., the top wall. As expected, wall temperature decreases along the boiler, while gas is transferring heat to the working fluid and, to a lesser extent, to the environment by free convection and radiation. Figure 12b shows the vertical paths on the gas side. Notice the wavy pattern of wall temperature, which is easily identified too in a visual analysis of the thermal image in Figure 10.

Although the geometry of the boiler is almost symmetric, wall temperatures in the vertical paths of Figure 12a are not as symmetric as would be expected, being seriously displaced in every case. This is likely to be mainly due to the perspective of the camera with regard to the boiler, which is not orthogonal. The position of the camera was a compromise solution between having the most information possible in a single image and making the process of taking the images as automated and standardized as possible, with no need to change the camera or boiler position twice in every experiment.

**Figure 13:**
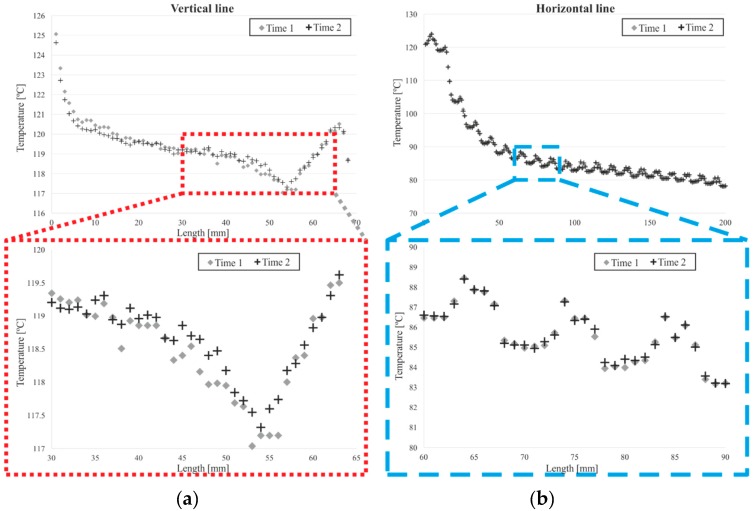
Comparison of temperature measurements following the methodology, in the same line, at two different moments. Analysis of a vertical line (**a**), and a horizontal line (**b**).

In order to evaluate the inaccuracies regarding the methodology developed, the depicted thermographic results show the range of temperatures quantified within paths that spanned five pixels in width. Moreover, in Figure 13, the temperatures measured at two different moments, following the same methodology and the same lines—one vertical and one horizontal—are compared. The error obtained was lower than the uncertainty of the camera in all the lines tested.

## 5. Conclusions

In this study, an experimental bench was presented to test boilers for an Organic Rankine Cycle in a waste heat recovery system, as well as a methodology to detect and analyze them via pattern recognition with thermography. This methodology is able to handle a large number of thermal images in a database and automatically extract paths of wall temperatures. These paths could be useful for developing correlations that can be used in predictive (zero-dimensional, one-dimensional, or computational fluid dynamics (CFD)) models.

Once the thermal images were analyzed, phase transition on the working fluid side was clearly noticeable, identifiable by a stage where wall temperature remained almost constant, in contrast with the remaining length of the path.

Future work will focus on the experimental set-up, principally by carrying out further analysis of pressure drop in working fluids, and in the thermographic analysis, mainly by comparing the results obtained with a CFD solution and diving deeper into the working fluid phase transition.

## Figures and Tables

**Table 1:** Main components of the test bench. WF—working fluid; AC—alternating current.

	Components	Remarks
Air circuit	Air blower	Power: 4 W Voltage: 345–415 V Maximum flow rate: 318 m^3^/h Maximum pressure drop: 390 mbar
	Air heating	Atmospheric pressure Made of a resistance of 27 kW at 240 V AC Maximum temperature: 750 °C
WF circuit	Pump (ethanol)	Power: 1.5 kW Maximum flow rate: 50 L/h Maximum speed: 233 rpm
	Condenser	Heat exchanger with plates Ethanol refrigerated with water
Refrigerant circuit	Pump (water)	Flow rate: 3.25 L/h Speed: 2820 rpm Power: 0.37kW
	Heating unit	Maximum air flow rate: 4080 m^3^/h Refrigeration capacity: 20.1 kW Heating capacity: 30.7 kW Maximum water flow rate: 57.6 L/min

**Table 2:** Description of the main sensors of the test bench.

Sensor	Remarks	Uncertainty
Air	Sensor K	1.5 °C
Water/ethanol	Sensor T	0.5 °C
Pressure drop	Differential pressure transducer	0.4%
Mass flow rate	Coriolis mass flow meter	0.11%

**Table 3:** Main features of the thermographic camera used.

Model	Flir E60
Range of temperature	−20 °C to 650 °C
Thermal sensitivity	<0.05° to 30°
Resolution	320 × 240 pixels

**Table 4:** Boiler operating conditions.

	Ethanol	Dry Air
Inlet mass flow (kg/h)	30	70
Inlet temperature (°C)	80	700
Pressure outlet (bar-a)	21	1
Outlet temperature (°C)	275.14	131.44
Performance (%)	85.3	

## References

[1] Domingues A., Santos H., Costa M. (2013). Analysis of vehicle exhaust waste heat recovery potential using a Rankine cycle. Energy.

[2] Park T., Teng H., Hunter G.L., van der Velde B., Klaver J. (2011). A Rankine Cycle System for Recovering Waste Heat from HD Diesel Engines—Experimental Results. SAE Int..

[3] Wang E.H., Zhang H.G., Fan B.Y., Ouyang M.G., Zhao Y., Mu Q.H. (2011). Study of working fluid selection of organic Rankine cycle (ORC) for engine waste heat recovery. Energy.

[4] Wang T., Zhang Y., Peng Z., Shu G. (2011). A review of researches on thermal exhaust heat recovery with Rankine cycle. Renew. Sustain. Energy Rev..

[5] He M., Zhang X., Zeng K., Gao K. (2011). A combined thermodynamic cycle used for waste heat recovery of internal combustion engine. Energy.

[6] Horst T.A., Rottengruber H.S., Seifert M., Ringler J. (2013). Dynamic heat exchanger model for performance prediction and control system design of automotive waste heat recovery systems. Appl. Energy.

[7] Saleh B., Koglbauer G., Wendland M., Fischer J. (2007). Working fluids for low-temperature organic Rankine cycles. Energy.

[8] Schmid H. Less Emission Through Waste Heat Recovery. Proceedings of the Green Ship Technology Conference.

[9] Teng H. (2010). Waste Heat Recovery Concept to Reduce Fuel Consumption and Heat Rejection from a Diesel Engine. SAE Int..

[10] Silva J.J.D., Maribondo J.F. (2018). Analysis of lubricating oils in shear friction tests using infrared thermography. Infrared Phys. Technol..

[11] Liu T.L., Pan C. (2016). Infrared thermography measurement of two-phase boiling flow heat transfer in a microchannel. Appl. Therm. Eng..

[12] Hetsroni G., Mosyak A., Segal Z., Ziskind G. (2002). A uniform temperature heat sink for cooling of electronic devices. Int. J. Heat Mass Transf..

[13] Xu J.L., Zhang W., Wang Q.W., Su Q.C. (2006). Flow instability and transient flow patterns inside intercrossed silicon microchannel array in a micro-timescale. Int. J. Multiph. Flow.

[14] Li H., Hrnjak P. (2015). Quantification of liquid refrigerant distribution in parallel flow microchannel heat exchanger using infrared thermography. Appl. Therm. Eng..

[15] Leblay P., Henry J.F., Caron D., Leducq D., Bontemps A., Fournaison L. Infrared Thermography applied to measurement of Heat transfer coefficient of water in a pipe heated by Joule effect. Proceedings of the 11th Quantitative InfraRed Thermography.

[16] Carlomagno G.M., Cardone G. (2010). Infrared thermography for convective heat transfer measurements. Exp. Fluids.

[17] Scaccabarozzi R., Tavano M., Invernizzi C.M., Martelli E. (2018). Comparison of working fluids and cycle optimization for heat recovery ORCs from large internal combustion engines. Energy.

[18] Duda R.O., Hart P.E. (1972). Use of the Hough Transformation to Detect Lines and Curves in Pictures. Commun. ACM.

[19] Hough P.V.C. (1962). Method for Recognizing Complex Patterns. U.S. Patent.

[20] Torrente M.L., Biasotti S., Falcidieno B. (2018). Recognition of feature curves on 3D shapes using an algebraic approach to Hough transforms. Pattern Recogn..

